# Impulsivity and Childhood Trauma Experience in Borderline Personality Disorder and Healthy Controls: A Comparative Study

**DOI:** 10.31729/jnma.9100

**Published:** 2025-07-01

**Authors:** Pratistha Ghimire, Sulochana Joshi, Rabi Shakya

**Affiliations:** 1 Paropakar Maternity and Women's Hospital, Thapathali, Kathmandu, Nepal; 2 Department of Psychiatry, Patan Academy of Health Sciences, Lalitpur, Kathmandu, Nepal

**Keywords:** *Borderline personality disorder*, *childhood trauma*, *impulsivity*

## Abstract

**Introduction:**

Borderline personality disorder is a chronic psychiatric disorder characterized by pervasive patterns of affective instability, self-image disturbances, interpersonal relationship instability, marked impulsivity and suicidal behaviour. Impulsive trait is a major component of BPD. Another major risk factor for the development of borderline personality disorder is childhood trauma. The objective of this study was to compare childhood trauma and impulsivity in borderline personality disorder and healthy controls.

**Methods:**

This is a hospital based cross sectional comparative study. Patients seeking treatment in inpatient and outpatient in Department of psychiatry, Patan Hospital, Lalitpur, Nepal who fulfilled the inclusion and exclusion criteria were taken. Patients were divided into two groups: BPD (n=21) and HC (n=42). Childhood trauma questionnaire-28 and Barratt Impulsivity score-11 were filled.

**Results:**

The mean Barratt Impulsivity score-11 for borderline personality disorder was 76.95±11.06 and 66.42±8.92 in health controls. The observed difference was statistically significant (p=<0.001). Childhood trauma questionnaire-28 score for borderline personality disorder was 69.05±21.37 and that for healthy control was 46.43±9.27. The observed difference was statistically significant (p-value<0.05).

**Conclusions:**

In this study higher impulsivity and childhood trauma experience was observed with BPD than HC.

## INTRODUCTION

Borderline personality disorder (BPD) is a chronic psychiatric disorder characterized by pervasive patterns of affective instability, self-image disturbances, interpersonal relationship instability, marked impulsivity and suicidal behavior.^1^ Impulsivity is a predisposition toward rapid, unplanned reactions to internal or external stimuli without regard to negative consequences of these reactions to the impulsive individuals or to others.^2^ Impulsivity is a major component of BPD. Childhood trauma (CT) is defined as willingly or unwillingly behavior by an adult that adversely affects the child's physical and psychosocial development and causes long-term effects on the child.^3^

Limited published literature has studied impulsivity & childhood trauma experiences and assessed relationship between each other in BPD patients. There are scanty of published literature in this part of the world. The objective of this study is to compare childhood trauma experience and impulsivity between BPD and Healthy controls (HC).

## METHODS

This was a hospital based cross sectional comparative study. Patients seeking treatment in inpatient and outpatient in Department of psychiatry, Patan Hospital, Patan Academy of Health Sciences (PAHS), Lalitpur, Nepal who fulfilled the inclusion and exclusion criteria were taken. Duration of the study was 1 year. Approval of the study was obtained from the Institutional Review Committee of PAHS (Reference number: PMS2103161494). Informed written consent was obtained from all participants.

For written consent generic PAHS format in English and Nepali were used. Participants could withdraw from the study at any time without giving any reason during the study period. Confidentiality of the research participants was ensured. A statement indicating that the participants had understood all the information in the consent form and was willing to participate voluntarily were obtained. When required, a witness was present during the interview.

Patients were divided into two groups: BPD and HC. In BPD group, patients with borderline personality disorder as diagnosed as per diagnostic and statistical manual of mental disorders-5 (DSM-5) by consultant psychiatrist, patients within age range of 18-65 year, patients with known case of depressive disorder under remission, patients with known case of substance use disorder and patients willing to participate in the study and who gave informed written consent were included. While in HC group, age and gender matched and participants willing to participate in the study and who gave informed written consent were included. Patients who were unable/unwilling to give consent and patients diagnosed as other psychiatric disorders and other personality disorders (current as well as life time) by consultant psychiatrist were excluded.

Convenience sampling method was used. Sample size was determined using mean & standard deviation (SD) of childhood trauma in BPD and HC groups in a review study.^4^ Mean & Standard Deviation (SD) of childhood trauma in BPD was 51.7±18.8 and that of childhood trauma in HC was 34.4±12.7. At 80% power and 1% of level of significance and ratio of BPD: HC=1:2 sample size calculated for BPD patients were 21 and for HC were 42. So, the total sample size was 63.

Clinicodemographic proforma, Childhood trauma questionnaire-28 (CTQ-28) and Barratt Impulsivity score-11(BIS-11) were filled by two groups. Data was entered in Microsoft Excel and analyzed in EPI INFO and R. Shapiro-Wilk test applied to test normality. Clinico demographic data were analyzed using descriptive statistics. In between group comparisons were carried out. Correlation of CTQ and impulsivity scores in the two study groups (BPD and HC) was done. Level of statistical significance was kept at p<0 .05 for all the tests.

## RESULTS

A total of 21 BPD patients and 42 HC were taken. The mean age of BPD group was 21.09±3.71 years and that of HC was 20.40±4.33 years. There were 19 (90.48%) female in BPD group and 38 (90.48%) in HC group. Similarly male were 2(9.52%) in BPD group and 4(9.52%) in HC group.

Clinical profile of patients in BPD and HC results showed that family history of psychiatric illness was observed in 6 (28.57%) patients , past history of depressive disorder in 12 (57.14%), past history of selfharm 21(100%) ,past history of suicide attempt in 13 (61.09%) and past history of substance was observed in 10 (47.62%) patients (Table 1).

**Table 1:** Clinical profile of patients in BPD and Healthy Controls

Variables	BPD(n=21) n(%)	HC (n=42) n(%)
Family History of Psychiatric Illness	6(28.57%)	2(4.76%)
Past History of Depressive disorder	12(57.14%)	1(2.38%)
Past History of Self Harm	21(100%)	4(9.52%)
Past History of Suicide attempt	13(61.30%)	0(0%)
Past History of Substance Use	10(47.62%)	1(2.38%)
Under Any Treatment	19(95%)	0(0%)

The mean age of first self harm was 14.29+4.291 years in BPD group and that in healthy control group it was 16.50+2.517. No suicidal attempt or hospital admission for self harm or suicidal attempt was observed in HC group. However, in BPD group mean age of first suicidal attempt was 17+4.991 years and mean hospital admission for self harm or suicidal attempt was 1.71+1.496 times.

The total BIS-11 score in BPD group was 76.95±11.06 and that in HC group was 66.42±8.92 (Table 2). The mean CTQ-28 score for BPD was 69.05±21.37 and that for HC was 46.43±9.27 (Table 3). Independent T test was conducted to compare total BIS-11 score of BPD and HC. There was significant difference in impulsivity with total BIS score of BPD (M=76.95, SD=11.06) and healthy control (M=66.42, SD=8.92555) and p=<0.001. While comparing subscales motor impulsiveness and non-planning impulsiveness were statistically significant with P value <0.001 and 0.04 respectively. Attentional impulsiveness was not statistically significant (Table 4).

Mann Whitney U test was used to compare Total CTQ-28 scores in BPD and HC. The mean ranks of total CTQ-28 score of BPD=46 and healthy control = 24.85. There is statistical significance in total CTQ-28 score (p-value<0.05) between BPD and healthy control. Subscales scores as emotional abuse, sexual abuse, physical abuse, emotional neglect, physical neglect were higher in BPD and HC (Table 5).

**Table 2:** BIS-11 Score of patients in BPD and Healthy Control (n=63).

Variables	BPD (n=21)		Healthy Controls(n=42)	
	Mean±SD	Range	Mean±SD	Range
Total BIS Score	76.95±11.06	53-96	66.42±8.92	50-85
Subscales				
Attentional Impulsiveness	13.76±3.20	9-20	13.95±3.02	7-22
Motor Impulsiveness	26.76±6.33	17-39	19.60±3.37	11-26
Non Planning Impulsiveness	28.10±5.32	17-38	25.19±5.13	13-37

**Table 3:** CTQ-28 Score of patients in BPD and Healthy Controls (n=63).

Variable	BPD (n=21)		Healthy Controls(n=42)	
	Mean±SD	Range	Mean±SD	Range
Total CTQ score	69.05±21.37	41-102	46.43±9.27	36-76
Subscales				
Emotional Abuse	16.00±6.21	5-25	7.33±3.31	5-18
Physical Abuse	13.05±6.84	5-25	6.33±2.70	5-19
Sexual Abuse	10.57±5.07	4-20	6.45±2.63	5-15
Emotional Neglect	15.76±5.34	6-25	8.86±3.17	5-17
Physical Neglect	10.48±3.99	5-20	6.55±2.50	5-16

**Table 4:** Comparison of BIS-11 Score of patients in BPD and Healthy Controls (n=63).

Variables	BPD (n=21) Mean±SD	Healthy Control(n=42) Mean±SD	Mean Difference	95% CI of Mean		P value
				Lower	Upper	
Total BIS Score	76.95±11.06	66.42±8.92	10.52	5.35	15.69	<0.001
Subscales						
Attentional Impulsiveness	13.76±3.20	13.95±3.02	-0.19	-1.84	1.45	0.85
Motor Impulsiveness	26.76±6.33	19.60±3.37	7.16	4.72	9.60	<0.001
Non Planning Impulsiveness	28.10±5.32	25.19±5.13	2.90	0.12	5.68	0.04

**Table 5:** Comparison of CTQ-28 Score of patients in BPD and Healthy Controls (n=63).

Variables	BPD (n=21) (Mean rank)	Healthy Controls (n=42) (Mean rank)	Mann Whitney U Test		
			U	Z	P value
Total CTQ Score	46.31	24.85	140.50	-4.385	<0.001
Subscales					
Emotional Abuse	42.34	24.88	100	-5.05	<0.001
Physical Abuse	45.79	25.11	151	-4.41	<0.001
Sexual Abuse	40.81	20.61	256	-2.89	<0.001
Emotional Neglect	47.26	24.37	120	-4.69	<0.001
Physical Neglect	44.79	25.61	172.50	-4.06	<0.001

## DISCUSSION

In our study patients with BPD were found to have high levels of impulsivity. The higher level of impulsivity supports previous findings.^5^ Comparison of impulsivity between BPD and HC showed significant results. However, a study done in UK by Barker et al in 2015 found no significant difference in impulsivity between BPD and HC.^6^ This contrasts with our findings. The study done in UK included HC from community which could have had a volunteer bias. Severity of BPD symptoms correlates with impulsivity as measured by the BIS-11.^7^ BPD population in that study could have represented patient experiencing less severe BPD symptoms as patients from only out-patient were recruited but we recruited from out-patient, emergency and in-patient facilities.

BIS-11 subscales as motor and non-planning impulsiveness scores were higher in BPD than HC. However, attentional impulsiveness was comparable between two groups. While comparing the subscales there was statistically significance with motor and non-planning impulsiveness. However, attentional impulsiveness did not show statistically significant result. Previous studies comparing subscales have shown significant result.^6,8^

Non-planning impulsiveness was defined by Patton et al as a difficulty with planning actions carefully and thinking about consequences of actions.^9^ Our study population was recruited mostly from emergency department or out-patient clinic either with self-harm or externalizing aggressive behaviour. Therefore, these patients may have scored higher in motor impulsiveness and non-planning impulsiveness. Motor control may be related to what is generally referred to as behavioural disinhibition, a trait present in BPD which was significant in our BPD patients.^9^ This presenting symptom may not have been captured by attentional impulsiveness subscale thus showing no significant difference than HC. Our findings highlight the importance of setting of study and the disadvantage of using self-reported questionnaires of impulsivity over behavioural measures as in other study done in 2010.^10^

In our study childhood trauma experience was higher in BPD group. This is consistent with previous study done by Laporte et al in Montreal in 2011.^11^ While comparing childhood trauma experience between BPD and HC the result was statistically significant. In a similar study childhood trauma experience were higher in BPD than in HC. This study revealed that emotional abuse, sexual abuse, physical abuse, emotional neglect, physical neglect was higher in BPD than HC. Similar findings about various domains of childhood trauma were shown in previous study.^11^ While comparing emotional abuse, sexual abuse, physical abuse, emotional neglect, physical neglect between BPD and HC our study showed statistical significance.

Five studies independently found an association between physical abuse and BPD.^12-16^ Guzder et al found that physical abuse was not common in children with BPD.^14^ This was in contradiction to our findings. The sample populations in that study were children aged between 7-12 years. The diagnosis was done using Diagnostic Interview for Borderlines (C-DIB-R). The difference in assessment by different tools, recall bias and under reporting by parents could be the reason for difference in findings. Three studies by Guzder et al in 1998, Zelkowitz et al in 2001 and Guzder et al in 1999 found an independent association between sexual abuse and BPD.^15,17,18^ One study by Goldman et al in 1992 found that sexual abuse was not more common in children with BPD.^13^ In study done by Goldman et al history of sexual abuse was obtained from both the children and families as part of a general psychiatric evaluation in which abuse was not the referring complaint. Abusive families tend to be secretive about these activities and children are often forced into silence.^18^ Under reporting of sexual abuse by parents could have been another reason for difference in results. Hecht et al revealed that children with higher levels of borderline features did not have higher levels of emotional abuse.^15^ These studies used participants who were children between 6-12 years and 10-12 years respectively who attended a summer camp research program. BPD precursor composite was examined in former study and Borderline personality feature scale for children (BPFS-C) was used to assess BPD features in latter study. BPFS-C is a self-report questionnaire.^19^ This could be a reason for difference in the results as this study only examined the BPD precursors that led to development of BPD in these children. It may not have represented the true BPD population unlike our study. Two studies by Guzder et al and Hecht et al had higher level of neglect in BPD which was similar to this study.^15,16^ However these studies did not look upon emotional and physical neglect separately.

Childhood maltreatment can have devastating effects on the development of healthy and adaptive emotion regulation and self-control.^20^ Previous studies have reported positive relationships between trauma and impulsivity. Ruggiero, Bernstein & Handlesman revealed that childhood trauma were significantly associated with impulsive personality disorders in male veterans.^21^ Johnson et al reported similarly in a non-veteran population.^22^ However the study done by Lepouriel et al showed that when CTQ-28 and BIS-11 score correlation was assessed there was no statistical significance.^4^

As per the literature review and our findings, we can assume that BPD patients maybe inherently impulsive and have high childhood trauma. Furthermore, unfavorable childhood experiences may make BPD patients more impulsive.

This study has several limitations. Sampling method used in this study was convenience sampling. Impulsivity was measured using a self-report questionnaire rather than an objective examination. Similarly, there was no validated scale in Nepali language. Scales as International Personality Disorder Examination (IPDE) were not used for aiding in diagnosis of BPD and only clinical diagnosis was done. This was a single-center study, so the findings of this study could not be generalized to the entire population of the country and may not reflect the actual scenario. The fact that we had participants (BPD) who were receiving psychotropics is another drawback. The other drawback of the current study is that it assessed childhood histories of abuse retrospectively. The results might be impacted by the potential for retrospective memory bias. So, a longitudinal study will be required to verify the findings.

## CONCLUSIONS

In this study higher impulsivity was seen in BPD than HC. Patient with BPD experienced more childhood trauma experience than HC.

## References

[1] Lieb K, Zanarini MC, Schmahl C, Linehan MM, Bohus M (2004). Borderline Personality Disorder.. The Lancet..

[2] Moeller FG, Barratt ES, Dougherty DM, Schmitz JM, Swann AC (2001). Psychiatric Aspects of Impulsivity.. Am J Psychiatry..

[3] Quinn M, Caldara G, Collins K, Owens H, Ozodiegwu I, Loudermilk E (2018). Methods for Understanding Childhood Trauma: Modifying the Adverse Childhood Experiences International Questionnaire for Cultural Competency.. Int J Public Health..

[4] Richard-Lepouriel H, Kung AL, Hasler R, Bellivier F, Prada P, Gard S (2019). Impulsivity and Its Association With Childhood Trauma Experiences Across Bipolar Disorder, Attention Deficit Hyperactivity Disorder and Borderline Personality Disorder.. J Affect Disord..

[5] Moeller FG, Barratt ES, Dougherty DM, Schmitz JM, Swann AC (2001). Psychiatric Aspects of Impulsivity.. Am J Psychiatry..

[6] Barker V, Romaniuk L, Cardinal RN, Pope M, Nicol K, Hall J (2015). Impulsivity in Borderline Personality Disorder.. Psychol Med..

[7] Fossati A, Barratt ES, Carretta I, Leonardi B, Grazioli F, Maffei C (2004). Predicting Borderline and Antisocial Personality Disorder Features in Nonclinical Subjects Using Measures of Impulsivity and Aggressiveness.. Psychiatry Res..

[8] Silk KR (2010). The Quality of Depression in Borderline Personality Disorder and the Diagnostic Process.. J Personal Disord..

[9] Patton JH, Stanford MS, Barratt ES (1995). Factor Structure of the Barratt Impulsiveness Scale.. J Clin Psychol..

[10] Jacob GA, Gutz L, Bader K, Lieb K, Tuscher O, Stahl C (2010). Impulsivity in Borderline Personality Disorder: Impairment in Self-Report Measures, but Not Behavioral Inhibition.. Psychopathology..

[11] Laporte L, Paris J, Guttman H, Russell J (2011). Psychopathology, Childhood Trauma, and Personality Traits in Patients With Borderline Personality Disorder and Their Sisters.. J Personal Disord..

[12] Belsky DW, Caspi A, Arseneault L, Bleidorn W, Fonagy P, Goodman M (2012). Etiological Features of Borderline Personality-Related Characteristics in a Birth Cohort of 12-Year-Old Children.. Dev Psychopathol..

[13] Goldman SJ (1992). Physical and Sexual Abuse Histories Among Children With Borderline Personality Disorder.. Am J Psychiatry..

[14] Guzder J, Paris J, Zelkowitz P, Marchessault K (1996). Risk Factors for Borderline Pathology in Children.. J Am Acad Child Adolesc Psychiatry..

[15] Hecht KF, Cicchetti D, Rogosch FA, Crick NR (2014). Borderline Personality Features in Childhood: The Role of Subtype, Developmental Timing, and Chronicity of Child Maltreatment.. Dev Psychopathol..

[16] Winsper C, Zanarini M, Wolke D (2012). Prospective Study of Family Adversity and Maladaptive Parenting in Childhood and Borderline Personality Disorder Symptoms in a Non-Clinical Population at 11 Years.. Psychol Med..

[17] Guzder J, Paris J, Zelkowitz P, Feldman R (1999). Psychological Risk Factors for Borderline Pathology in School-Age Children.. J Am Acad Child Adolesc Psychiatry..

[18] Krause-Utz A, Sobanski E, Alm B, Valerius G, Kleindienst N, Bohus M (2013). Impulsivity in Relation to Stress in Patients With Borderline Personality Disorder With and Without Co-Occurring Attention-Deficit/Hyperactivity Disorder: An Exploratory Study.. J Nerv Ment Dis..

[19] Nelson DA, Coyne SM, Swanson SM, Hart CH, Olsen JA (2014). Parenting, Relational Aggression, and Borderline Personality Features: Associations Over Time in a Russian Longitudinal Sample.. Dev Psychopathol..

[20] Young JC, Widom CS (2014). Long-Term Effects of Child Abuse and Neglect on Emotion Processing in Adulthood.. Child Abuse Negl..

[21] Ruggiero J, Bernstein DP, Handelsman L (1999). Traumatic Stress in Childhood and Later Personality Disorders: A Retrospective Study of Male Patients With Substance Dependence.. Psychiatr Ann..

[22] Johnson JG, Cohen P, Brown J, Smailes EM, Bernstein DP (1999). Childhood Maltreatment Increases Risk for Personality Disorders During Early Adulthood.. Arch Gen Psychiatry..

